# Does my lifestyle explain my depression? The role of exercise, diet and smoking in the prevention of depression

**DOI:** 10.1192/j.eurpsy.2022.1751

**Published:** 2022-09-01

**Authors:** R. Gomes, C. Santos, N. Descalço, F. Moutinho

**Affiliations:** Hospital Garcia de Orta, Psychiatry, Almada, Portugal

**Keywords:** Depression, exercise, smoking, diet

## Abstract

**Introduction:**

Depression as a public health concern highlights the importance of prevention. The nature of the disease is complex, linked to numerous biopsychosocial factors. However, it was found that healthiest lifestyle reduced 67% the risk of depressive symptoms.

**Objectives:**

To review evidence on how exercise, diet, and smoking impact on the risk of depression.

**Methods:**

Non-systematic review of literature through search on PubMed/MEDLINE following the terms “Lifestyles”,“risk” and “depression”.

**Results:**

Several studies have shown that exercise reduces the incidence of depressive symptoms and major depressive disorder regardless of intensity, geographic region, age, gender, or follow-up period. Smoking significantly increases the risk of depression, including the ones exposed to second-hand smoking and pregnant women in which prenatal smoking was associated with a three-fold increased risk of postpartum depression. The Mediterranean diet rich in complex carbohydrates, omega-3 fatty acids, B-group vitamins and several amino acids have shown a negative association with the incidence of depression. A high frequency of breakfast consumption, an increased intake of fruits, vegetables, and some specific nutrients (zinc, iron, magnesium, vitamins, and folate) was also inversely correlated with prevalence of depressive symptoms. On the other hand, western dietary patterns, with sweetened beverages, processed food, and foods rich in saturated fatty acids, have been linked to an increased risk. Skipping meals and snacking on unhealthy food also contributes to depressive symptoms.

**Conclusions:**

Relatively modest changes in population diet, tobacco consumption and levels of exercise may have important public mental health benefits preventing a substantial number of new cases of depression.

**Disclosure:**

No significant relationships.

